# Recent Developments in Spectroscopic Techniques for the Detection of Explosives

**DOI:** 10.3390/ma11081364

**Published:** 2018-08-06

**Authors:** Wei Zhang, Yue Tang, Anran Shi, Lirong Bao, Yun Shen, Ruiqi Shen, Yinghua Ye

**Affiliations:** Department of Applied Chemistry, School of Chemical Engineering, Nanjing University of Science and Technology, Nanjing 210094, China; tangyue090@163.com (Y.T.); 114103000380@njust.edu.cn (A.S.); blr1216@njust.edu.cn (L.B); 315103002440@njust.edu.cn (Y.S.); rqshen@njust.edu.cn (R.S.); yyinghua@njust.edu.cn(Y.Y.)

**Keywords:** explosive detection, terahertz spectroscopy, laser-induced breakdown spectroscopy, Raman spectroscopy, ion mobility spectrometry

## Abstract

Trace detection of explosives has been an ongoing challenge for decades and has become one of several critical problems in defense science; public safety; and global counter-terrorism. As a result, there is a growing interest in employing a wide variety of approaches to detect trace explosive residues. Spectroscopy-based techniques play an irreplaceable role for the detection of energetic substances due to the advantages of rapid, automatic, and non-contact. The present work provides a comprehensive review of the advances made over the past few years in the fields of the applications of terahertz (THz) spectroscopy; laser-induced breakdown spectroscopy (LIBS), Raman spectroscopy; and ion mobility spectrometry (IMS) for trace explosives detection. Furthermore, the advantages and limitations of various spectroscopy-based detection techniques are summarized. Finally, the future development for the detection of explosives is discussed.

## 1. Introduction

**Figure 1:**
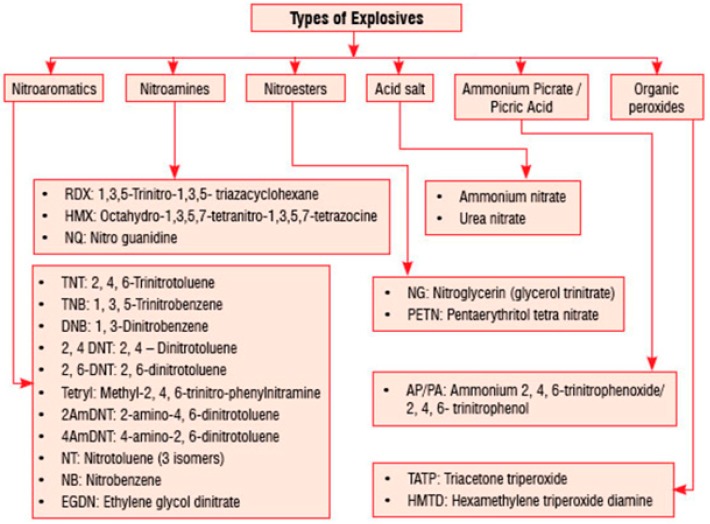
Classification of commonly used explosives.

Explosives and their related substances are widely used in many military conflicts and for varying civil applications, resulting in different explosives that have been synthesized and manufactured [1]. Figure 1 shows the classifications of the most commonly used explosives. 

In recent years, the detection of different kinds of energetic materials has become a high priority problem for anti-terrorism reasons [2,3]. It is also crucial to the protection of human life, infrastructure, and property [4]. Law enforcement forces of different countries around the world need to promote the development of effective detection systems to deal with the problem of concealment of explosives in public places such as airports, railways, or bus stations. Moreover, if terrorist attacks or crimes are successful, the development of an analytical tool to identify the remains of explosives is very important for the forensic rebuild at the site of the explosion [5]. In addition, water contamination, soil pollution, and health problems caused by explosive explosions should be taken into account. Based on these reasons, people need to develop faster and more sensitive explosives analysis methods [6,7]. 

Due to the importance of rapid, automatic, and non-contact detection of explosives for homeland security and environmental safety [8], a variety of spectroscopic technologies have been employed to detect trace quantities of explosives; for example, terahertz (THz) spectroscopy [9,10], laser induced breakdown spectroscopy (LIBS) [11,12,13,14,15,16], Raman spectroscopy [17,18,19,20,21,22], ion mobility spectrometry (IMS) [23,24,25,26], nuclear magnetic resonance (NMR) [27,28,29,30], nuclear quadrupole resonance (NQR) [31,32,33], laser-induced thermal emissions (LITE) [34,35], infrared (IR) spectroscopy [36,37,38], mass spectrometry [39,40,41,42,43,44,45,46], optical emission spectroscopy (OES) [47,48], photo-thermal infrared imaging spectroscopy (PT-IRIS) [49,50,51], photoacoustic techniques [52,53,54], FT-FIR spectroscopy [55], microwave [56], and millimeter-wave [57], etc. Various electromagnetic radiations such as X-ray [58] and γ rays [59] have also been employed in explosive detection. Each one of these techniques, however, has some specific limitations.

The applications of spectroscopy-based methods for trace explosives detection has progressed significantly in the past several years. The purpose of this review is to summarize the applications of terahertz spectroscopy, laser-induced breakdown spectroscopy, Raman spectroscopy, and ion mobility spectroscopy for the detection of explosives, and summarize future trends in their development.

## 2. Terahertz Spectroscopy

**Figure 2:**
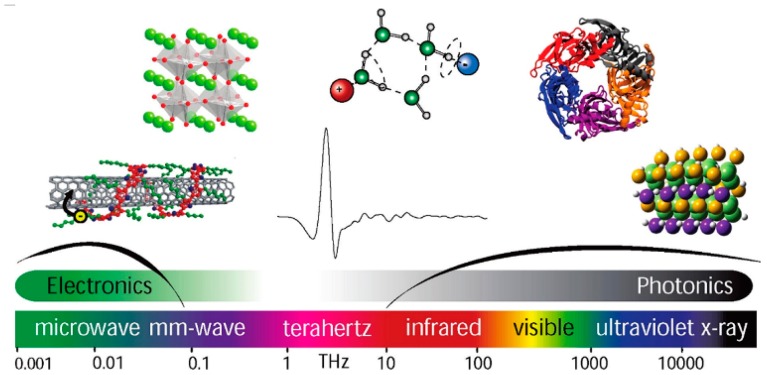
Electromagnetic spectrum in the THz range, with images highlighting the wide variety of molecules, materials, and phenomena that can be explored using THz spectroscopy. Adapted from [61], with permission from © 2011 American Chemical Society.

THz spectroscopy, with the electromagnetic radiation in the frequency range of 0.1–10 THz, can be used to detect different kinds of physical phenomena, such as rotation and vibration transitions for molecules, collective vibration or torsion modes for condensed-phase materials [60]. Electromagnetic spectrum in the THz range for different applications is schematically illustrated in Figure 2 [61]. 

The THz technology has the following advantages: it can transmit through a lot of common barrier materials, including packaging, plastics, paper, clothing, and ceramics, etc. These advantages make it possible to develop non-intrusive techniques that can detect hidden explosives. The lower related photon energy range is considered to be biologically safe, so the target being scanned is free from destructive photoionization. Explosives, drugs, and other biologics have unique absorption characteristics in the terahertz frequency range. [62,63,64] These spectral fingerprints are collectively due to the intermolecular and intramolecular vibrations [65,66]. The large-amplitude vibration modes detected in THz spectroscopy provide an in-depth understanding of the molecular structure dynamics by directly interrogating the motions responsible for conformational isomerization. Because of the delocalization property, the low frequency vibration mode is strongly influenced by the size and long-range order of molecules.

**Figure 3:**
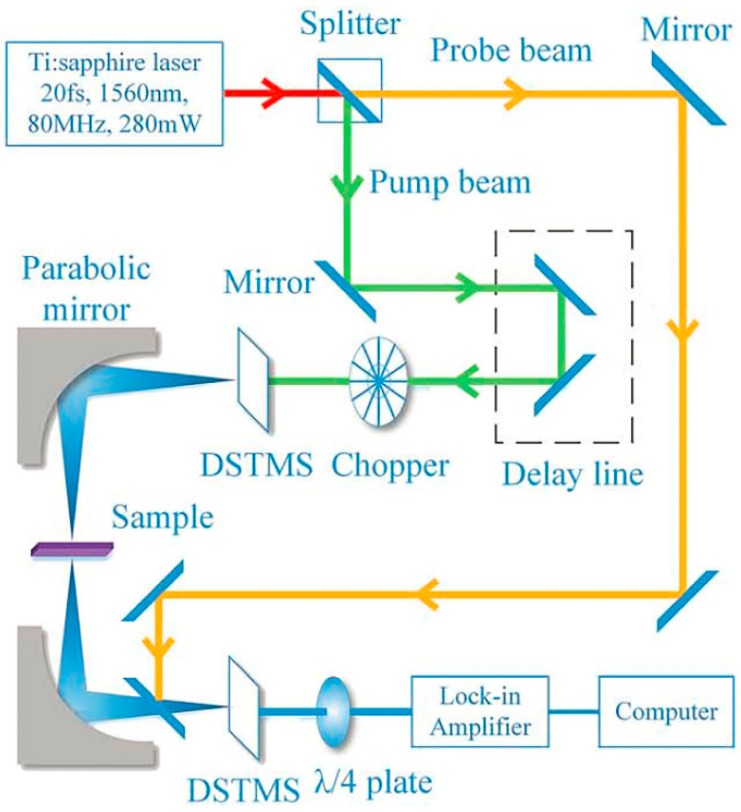
Schematic diagram of the transmission THz-TDS system. Adapted from [60], with permission from © 2016 Taylor & Francis.

Terahertz time domain spectroscopy (THz-TDS) can determine the complex permittivity of various specimen in a common frequency band of 0.2–3 THz, and upper frequencies can be reached with ultra-short laser pulses [61,67]. The THz electromagnetic radiation produced by the emitter can be detected by a receiver. The THz-TDS results relate to the real-time detection of the electric field vector of the THz electromagnetic wave after the transmission through the sample compartment [68]. A generic schematic of the TDS device is shown in Figure 3. The laser pulse is divided into the pump beam and probe beam by the splitter. The pump pulse is excited terahertz pulses that pass by the GaAs photoconductive antenna. After passing through the sample, the terahertz beam with the information of the sample then combine with the probe beam. The obtained weak signal is finally amplified by a lock-in amplifier and collected by a computer for final processing [60]. 

Sleiman et al. measured the absorption spectra of cyclotrimethylenetrinitramine (RDX), pentaerythritol tetranitrate (PETN), and their mixtures by THz-TDS. The samples were prepared by mixing pure explosives powder with polyethylene (PE). Preventing the overlapping of important spectral lines is a challenge for fingerprint inspection. A series of partial least squares regression models were built to predict the PETN concentrations in mixture samples. Moreover, when the original 0.2–3 THz (6–100 cm^−1^) spectral band was reduced to 1.8–3 THz (60–100 cm^−1^), the remarkably overlapping phenomenon of the spectral frequency band could be found. Furthermore, they revealed and identified specific explosives by applying partial least squares discriminant analysis to the absorbance THz images, and classifying each pixel as RDX, PETN, or RDX/PETN mixture. The identification and classification of explosives were successfully realized [69,70,71].

Trzcinski et al. reported the experimental results of the absorption spectra for RDX, PETN, and HMX by comparing with the absorption of their simulants using the THz-TDS system in the range of 0.1–3.0 THz. The samples contained 10% explosives and 90% Teflon. The absorbance peaks of all hazardous substances are identical in their properties, and their shapes are very similar. The oxidant measurement results show that THz measurement can be used not only for explosives detection but also for hazardous materials detection [72]. 

Pierno et al. obtained the terahertz spectra database of concealed PETN, RDX, TNT, and Semtex by common packaging materials. The sample in the form of polycrystalline was used without polypurification and diluted with polytetrafluoroethylene. The recorded terahertz spectrum has sufficient signal-to-noise ratio to identify hidden explosives. The detection limits for TNT is about 2% concentration by mass using broadband THz-TDS [66]. In another research, the samples of TNT, PETN, and RDX were prepared as 1 mg/mL solutions in methanol and characterized by THz-DS. The test results indicated that each temporal signal was distinctly different in intensity and shape, which showed the unique characteristics of the identified compounds [73]. The terahertz spectra of pure RDX, HMX, PETN, and their mixtures were also detected by using the TDS system at the transmission and reflection modes, the incident angle r was about 45◦ and the adopted distance was between stand-off with close to normal incidence with 30 and 40 cm. The absorbance peak value of all substances was related to the characteristics of reflectance [74].

**Figure 4:**
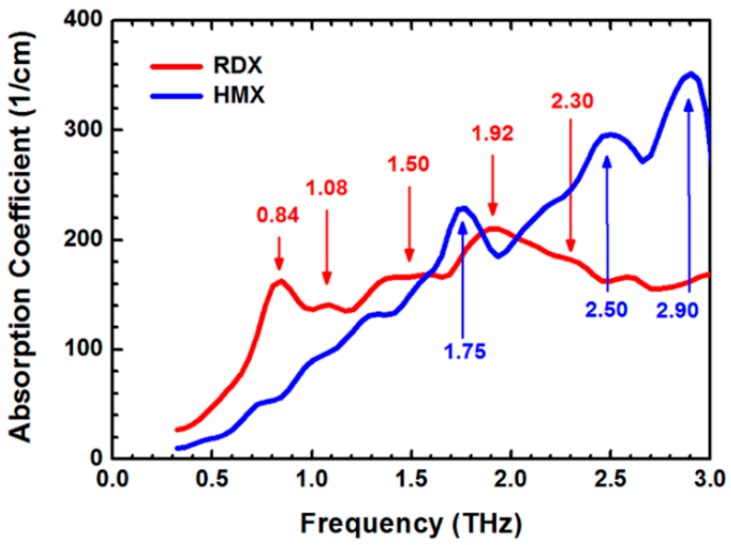
The absorption coefficient spectra of cyclotrimethylenetrinitramine (RDX) and HMX. Adapted from [75], with permission from © 2014 American Institute of Physics.

Reflection terahertz time-domain spectroscopy of nitramine explosives (RDX and HMX) were conducted in the range of 0.3–3 THz. Samples were prepared by pressing pure explosives that did not contain any additional binder or additives. The primary absorption peaks located at 0.84, 1.08, 1.50, 1.92, and 2.30 THz for RDX, and at 1.75, 2.50, and 2.90 THz for HMX, are shown in Figure 4. RDX and HMX had completely different spectra, which were beneficial to distinguish them [75]. 

For the purpose of providing references for the identification of high nitrogen compounds, Huang et al. measured terahertz spectra of 5-aminotetrazolium nitrate (5-ATN), hydrazine bistetrazolium-(4-methyl-1,5-diaminotetrazolium) (BMDATHBT), oxidizers (SrNO_3_, KClO_3_, KClO_4_), and mixtures in the range of 0.5–2.0 THz by using THz-TDS [76]. Theory calculations were also conducted by using the first principles method on the basis of density function theory (DFT) and the normal vibration modes of the crystal cells were performed by Dmol^3^. The measured and calculated resonance positions of these compounds are shown in Table 1. The two vibrating peaks for the 5-ATN/SrNO_3_ mixtures located at 0.72 THz and 1.23 THz are caused by 5-ATN and another peak at 1.80 is due to 5-ATN and SrNO_3_. Comparably, the vibration peaks at 1.55 THz and 1.67 THz are due to BMDATHBT while SrNO_3_ accounts for the peak at 1.80 THz for the BMDATHBT/SrNO_3_ composites. In other research, they also measured the absorption coefficient of 2,2′,4,4′,6,6′-hexanitrostillbene (HNS) with THz-TDS and Fourier-transform infrared spectroscopy (FTIR). The results showed that the characteristic peaks could be found at 1.7 THz and 3.1 THz. Both theoretical calculations and experimental results show that HNS has distinct characteristic peaks [77]. 

The characteristic THz-TDS absorption peaks of insensitive energetic material 2,6-diamino-3,5-dinitropyrazine-1-oxide (LLM-105) appeared in 1.27, 1.59, 2.00, 2.08, 2.20, and 2.29 THz. The DFT simulation results were in good agreement with the experimental results, with the exception of the 2.29 THz peak, which may be due to the lattice vibration or other reasons [78]. They also found the characteristic peaks for 1,1-diamino-2,2-dintroethylene (FOX-7) located at 1.59 and 2.12 THz [79]. For RDX, five main absorption peaks were located around 0.82, 1.05, 1.50, 1.92, and 2.20 THz, which agreed well with Choi’s results [75,80]. A subsequent study conducted by Guo et al., also demonstrated the use of THz-TDS to detect hexanitrohexaazaisowur-tzitane (ε-HNIW and γ-HNIW) in the 0.2–2.5 THz region. For ε-HNIW, the obvious absorption peaks could be found at 0.99, 1.32, 1.43, and 2.08 THz. For γ-HNIW, the primary absorption peaks appeared at 1.05, 1.52, and 1.90 THz [81]. 

Palka et al. measured the THz absorption spectra of six novel energetic materials: SAZ (azotetrazolatepentahydrat), AAZ (diammonium azotetrazolate), GUAZ (guanidinium azotetrazolate), TAGAZ (triamino-guanidiniumazo-tetrazolate), HNIW and TEX (tetraoxadinitroisowurtzitane) by using THz-TDS system [64]. All the materials were pressed into pellets for transmission investigations by mixing with PE powder, which is highly transparent in the THz region. The characteristic peaks of these explosives appeared in the range of 0.8–3 THz in room temperature. The absorption peaks for SAZ were at 1.34, 1.81, 2.35, 2.57, 2.75, and 2.92 THz. There are five absorption peaks for AAZ at 1.76, 2.40, 2.55, 2.71, and 2.86 THz. GUAZ could be distinguished by the peak at 1.38, 2.01, 2.31, 2.55, 2.84, and 2.91 THz. TAGAZ had the feature peaks at 1.10, 1.46, 1.67, 2.12, 2.25, 2.38, and 2.96 THz. The characteristic peaks of TEX could be found at 1.71, 2.33, 2.53, and 2.91 THz. The absorption peaks for HNIW (0.84, 1.45, 2.28, and 2.77 THz) are different than Guo’s results [81], which is due to the different crystalline forms of HNIW.

Choi et al. presented experimental results regarding principal interests and critical factors in the detection of explosive compounds by using THz-TDS. THz spectra of Composition B-3 and Pentolite, and suggested a novel signal processing method for in situ compound explosives detection [82]. Their signal processing procedure indicated that significant decrease was about 22.7% for Composition B-3 and 48.8% for Pentolite in noisy and humid environments.

Chrzanowski et al. applied THz-TDS to detect explosive liquids and liquid mixtures in analogy [83]. These explosive liquids can be significantly distinguished from each other on the basis of the refraction index in the lower THz region or by means of the absorption coefficient at a higher THz frequency range (1 THz).

Puc et al. studied the influence of different backgrounds for the spectral features of different explosive simulants by using THz-TDS [84]. Combined with the organic-crystal-based terahertz time-domain system and the spectral peak analysis method, the hidden simulants can be quickly detected and identified in the frequency region of 1.5 to 4.0 THz.

## 3. Laser-Induced Breakdown Spectroscopy

**Figure 5:**
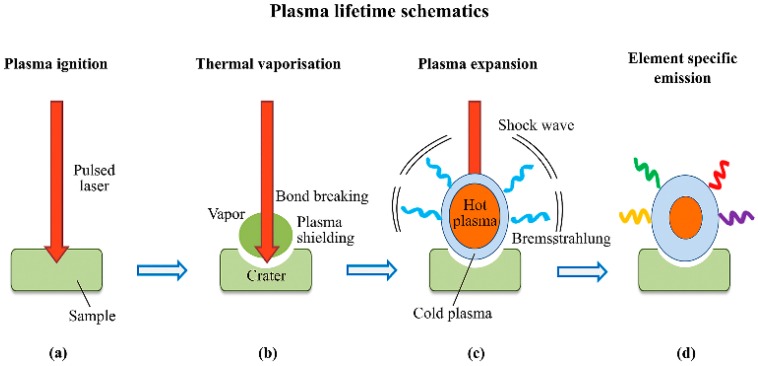
Main steps in the laser pulse/material interaction resulting in the generation of laser induced breakdown spectroscopy (LIBS) emission signals. (**a**) Plasma ignition; (**b**) thermal vaporization; (**c**) plasma expansion; (**d**) element specific emission. Adapted from [92], with permission from © 2017 Elsevier.

LIBS is an elemental and molecular fragment analysis and detection technology based on emission spectrometry and the laser shifts the emission from atom and molecule for detection [85]. During the last few years, the laser based technique has attracted a lot of attention for the detection of energetic compounds [86,87,88,89]. LIBS has been successfully applied to the analysis of elements and small molecular fragments in various materials. It is one of the potential technologies that can be used for explosive detection for the reasons of non-contact, rapid response, high sensitivity, real-time and multi-element detection characteristics. It has been employed for identification of explosive residues, chemical and biological agents on different surfaces such as polymers, and metals, etc. [90,91]. The main physical and chemical processes involved in LIBS are demonstrated in Figure 5 with reference to analysis of a solid target in air [92]. The analyte was first ablated, then atomized, and finally ionized to produce a plasma plume.

**Figure 6:**
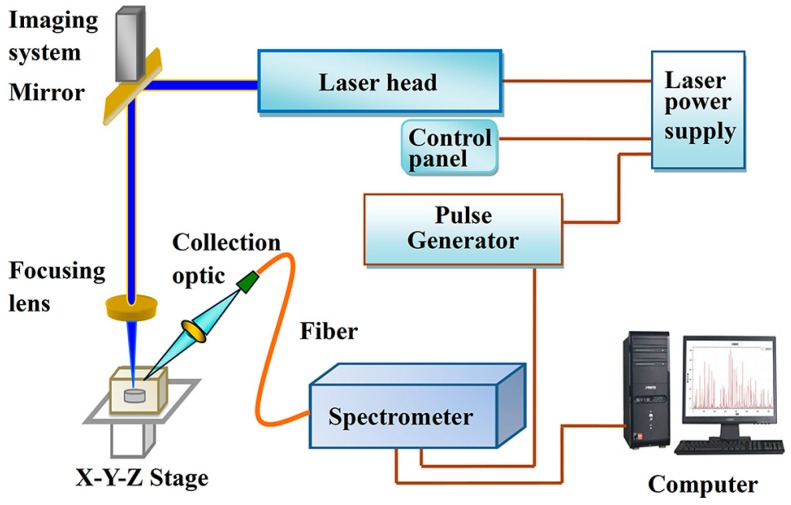
Schematic diagram of the LIBS experimental setup. Adapted from [97], with permission from © 2018 American Chemical Society.

LIBS has shown effective and promising results in the identification of explosive residues, the classification of polymers, and the analysis of complex biomaterials. It has been proven to be a real-time and effective chemical detection and analysis tool for the residue of energetic materials and explosives [93,94,95,96]. A typical schematic diagram of the LIBS experimental setup is shown in Figure 6 [97]. 

**Figure 7:**
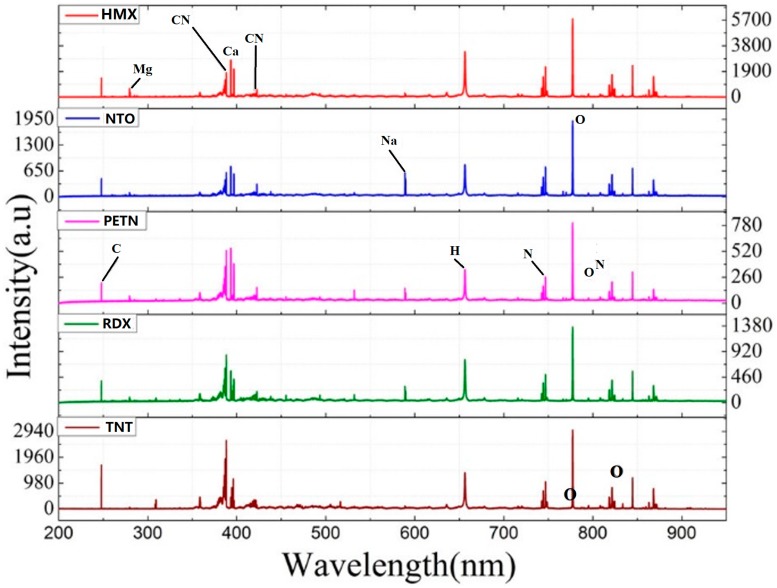
Representative LIBS spectra of typical explosives. Adapted from [98], with permission from © 2015 Springer Nature.

Myakalwar et al. measured the spectra of HMX, NTO, PETN, RDX, and TNT on their LIBS system, as shown in Figure 7 [98]. The emission peaks were associated with C (247.8 nm), Mg (279.5, 280.3 nm), Ca (393.3, 396.8, 422.7 nm), H (656.3 nm), N (742.4, 744.3, 746.9, 818.4, 818.8, 821.6, 824.2 nm), O (777.2, 777.4, 794.8, 822.2, 822.7, 844.6, 868.1 nm) and Na (589, 589.6 nm). Obviously, the peak with the highest intensity located at 777.3 nm corresponds to oxygen; the second strongest peak was found at 388.3 nm for CN, which was accompanied by the C_2_ peaks, indicating the production of organic molecules. 

Yang et al. performed IR-LIBS studies for ammonium perchlorate (AP), ammonium nitrate (AN), and ammonium sulfate (AS) samples under air and nitrogen atmospheric conditions. These three samples are commonly used as oxidants in explosives, showing obvious infrared emission characteristics from target molecules in the range of 4–12 μm. They also conducted the first study of mid infrared (MIR) LIBS of ammonium carbonate (AC). AC had distinct molecules/molecular debris infrared emission characteristics between 4 and 12 μm. The main emission peak near 4.4 μm could be attributed to the CO_2_ emission, which is caused by the sputtering carbon atom oxidation in the sample [99].

By using fs and ns pulses, Rao et al. recorded the laser-induced breakdown spectra of several explosive compounds (nitroimidazoles) in air and argon environments. Dominant atomic (C, H, N and O) and molecular (CN, C_2_ and NH) and emission lines were recognized [90]. The emission peak of CN was dominant in the air, while the emission characteristics of C_2_ were significant in the LIB spectra obtained in argon atmosphere.

The emission spectra from fs and ns laser-induced plasmas of nitrotriazolone (NTO), RDX, and HMX have been reported [100]. Their elemental and molecular characteristic spectra were compared. C and CN peaks exist in the LIBS spectra, which confirmed the characteristics of high energetic materials. Table 2 lists all the atomic and molecular peaks observed for the three explosives in the fs and ns LIBS spectra. Compared with the ns spectra, some of the Nitrogen peaks obtained in the fs LIBS spectra were weaker in intensity. The presence of an additional CN peak (at 385.01 nm) in the fs spectra was identified for all samples while in the ns spectra the peak was covered by the noise.

LIBS spectral characteristic lines for NTO, RDX, and TNT samples were obtained in air, nitrogen, and argon atmospheres by Sreedhar et al., as shown in Table 3 [101]. A slight change was observed depending on the nature of the sample and the surrounding atmosphere. The intensity of C (247.8 nm) and H_α_ (656.2 nm) peaks were strong in the argon condition for all the explosives. The TNT sample had a strong peak at C_2_ (516.47 nm) in an argon atmosphere. Compared with the spectra in argon, the CN violet band for *Δν* = 0 transitions in the spectral region of 385–388 nm was found to be strong in nitrogen and air conditions.

Rao et al. performed LIBS research on pyrazole, 1-nitropyrazole, 3-nitropyrazole, 3,4-dinitropyrazole and 1-methyl-3, 4,5 trinitro pyrazole [102,103]. CN molecular fragment bands in the ranges of 357–360 nm, 384–389 nm and 414–423 nm, C_2_ Swan bands in the range of 460–475 nm, 510–520 nm and 550–565 nm were observed. They also employed LIBS to investigate some nitropyrazole molecules in air, nitrogen, and argon atmospheres. The LIBS data proved the existence of molecular emissions of cyanide (CN) violet bands, diatomic carbon (C_2_) Swan bands, and atomic emission lines of C, H, O, and N. The observed decay times and molecular emission intensities were related to the number of nitro groups, atmospheric nitrogen content, and molecular oxygen balance.

Delgado et al. performed the LIBS characterization of TNT and PETN in N_2_ and air atmospheres [104]. The results indicated that C_2_ emission was strongly connected to the molecular structure as well as CN was the production of chemical reactions. In the H_2_ atmosphere, the results suggested that H_2_ might change the formation pathway of molecular fragments, thereby reducing the emission of CN and C_2_, and promoting the formation of NH, CH and OH. They have confirmed these results in an environment without air, where molecular fragment emissions were still very weak. This fact might be due to the reaction between H_2_/other atomic species and molecular species of the plasma leading to dissociation of small fragments (CN, C_2_, et al.).

De Lucia et al. obtained time-resolved LIBS spectra of some polymers and RDX and compared the atomic emission intensities of each sample [105]. It could be seen from the observed trend of the emission intensity that the atomic emission intensity may be related to the chemical composition of the sample. The relationship between emission intensity and molecular structure was a key factor to distinguish different kinds of energetic materials by LIBS. In addition, the plasma emission spectra of aluminized RDX explosives were obtained in air and argon atmosphere using LIBS technology. The atomic bands of Al, C, H, N, and O, and the molecular bands of AlO and CN have been determined. Aluminum and oxygen content in the plasma affected the produce mechanism of AlO and CN molecular bands [106].

**Figure 8:**
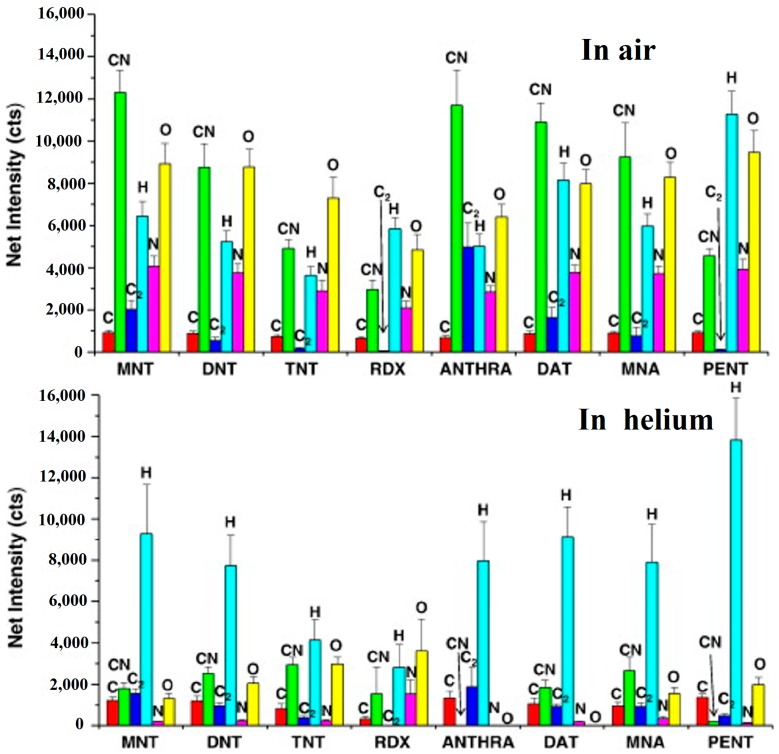
Net intensity of the LIBS signal in air and helium from several organic compounds. Adapted from [11], with permission from © 2011 Elsevier.

Aromatic nitrocompounds (MNT, DNT and TNT), RDX, anthracene, 2,4-diaminotoluene (DAT), 4-methyl-3-nitroaniline (MNA), and pentaerythritol (PENT) have been characterized by LIBS [11]. To prevent the secondary ionization and to distinguish between the spectral contribution owing to air from that of the substance in the plasma produced in air, the emission characteristics from atomic lines (C at 247.9 nm, H at 656.3 nm, N at 746.8 nm and O at 777.2 nm), and molecular bands (CN at 388.3 nm and C_2_ at 516.5 nm) have been researched in plasmas produced in air and in helium. As shown in Figure 8, significant differences in intensity distributions indicated that identification between compounds was possible. 

The LIBS spectra of nitrogen-rich compounds, such as 5-aminotetrazoliumni-trate (HAT-NO3) and hydrazinebistetrazole (HBT), bis(2,2,2-trinitroethyl)-hydrazodicarboxylate (BTHC), RDX, TNT, melamine, sucrose, and l-glutamine were recorded. The emission intensities of the atoms and the intensity ratios of the constituent elements in the LIBS spectrum are related to the mole fraction and the stoichiometry of the molecules. Moreover, the oxygen content in the molecule affects the emission intensity of molecular fragments, such as C2 [107].

Recently, standoff (up to ~2 m) and remote (~8.5 m) detection of new energetic compounds (nitroimidazoles and nitropyrazoles) were performed by using LIBS. Dominant spectral features of explosives (C, H, N, O atomic transitions and CN, C_2_, NH molecular bands) were readily identified [108]. 

## 4. Raman Spectroscopy

**Figure 9:**
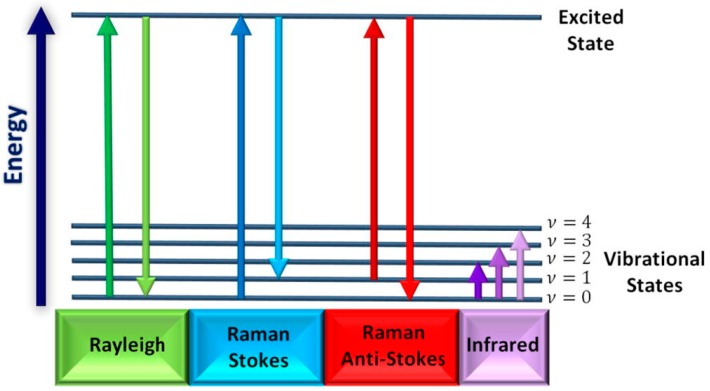
Modified energy diagram for Rayleigh and Raman scattering. Adapted from [109], with permission from © 2018 Elsevier.

As a vibrational spectroscopy, Raman spectra involve information about the specific arrangement and interaction of the atoms forming the molecule. It can be used to reveal the molecular information of a sample at the micro meter scale in a non-destructive way. Figure 9 describes the Rayleigh and Raman scattering [109]. Different from IR spectroscopy, Raman spectra show less dependent on the size of the particle and nature of the background surface. Raman spectra of different molecules have their unique fingerprint characteristics, that means each spectrum has specific and unique feature bands that can be selected for recognition [110]. 

**Figure 10:**
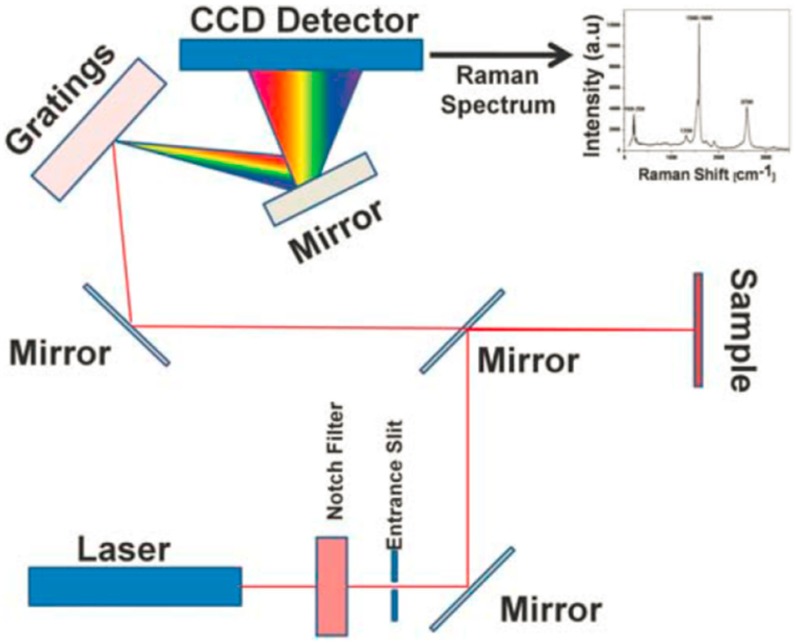
Schematic diagram of the Raman system. Adapted from [111], with permission from © 2017 Elsevier.

Due to the progress of instrument science, Raman spectroscopy technology is widely applied in various research fields, allowing scientists to extract new information from different samples. The information is highly molecularly specific and allows for a distinction between different types of explosives. The schematic of a Raman spectrometer is shown in Figure 10 [111]. After inducing by excitation laser shot on the sample, the Raman-scattered light of the sample was transferred to the spectrometer by using an optical fiber. The commonly used excitation laser sources are a 785 nm diode laser and 1064 nm Nd:YAG laser. 

**Figure 11:**
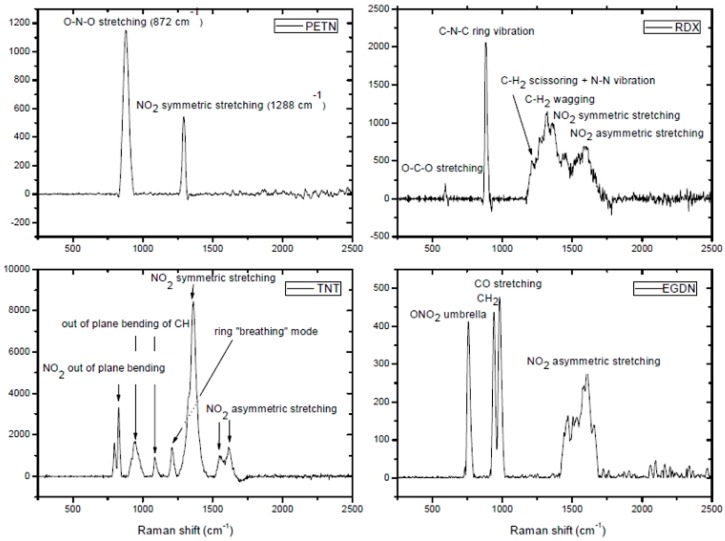
Surface-enhanced Raman spectra of PETN, RDX, TNT, and EGDN. Adapted from [112], with permission from © 2012 SPIE.

The Raman spectra of PETN, RDX, TNT, and ethane-1,2-diyl dinitrate (EGDN) in the spectral Raman shift range of 250–2500 cm^−1^ were obtained by Almaviva et al., as shown in Figure 11 [112]. Comparing the Raman spectra of these explosives, it was found that these substances can be easily identified by their main spectral characteristics, with strong characteristic peaks in the spectral wavenumber range of 250–1800 cm^−1^.

Jin et al. performed an experiment by using the homemade Raman standoff spectroscopy system to detect different explosives [113]. Raman spectra of TNT, RDX, HMX, PETN, and triacetone triperoxide (TATP) over a long distance up to 54 m were successfully recorded for explosive identification. Most of the shapes of feature peaks were preserved relatively well, and the chemical information could be well recognized despite some missing information, including the shifts and loss of some peaks of the observed Raman signal.

In another research, the UV resonance Raman spectroscopy/differential Raman cross-sections of TNT, PETN, RDX, HMX, and AN in acetonitrile and water solutions were measured at 204–257 nm. It was found that the spectral signal increases significantly with the decrease of excitation wavelength [114].

Hwang et al. measured the Raman spectra of representative energetic compounds (TNT, RDX, HMX, PETN, AN, NTO, HNIW, NQ, AND, AP, DMDNB, Tetryl, 2-DNT, and 4-ADNT) under the excitation wavelength of 514.5 nm, 632.7 nm, and 785 nm. Based on the measured spectral data, the relationship between the excitation wavelength, fluorescence effects, and their structure-property were revealed. The identification characteristics of different explosive compounds were obtained by principal component analysis [115]. 

**Figure 12:**
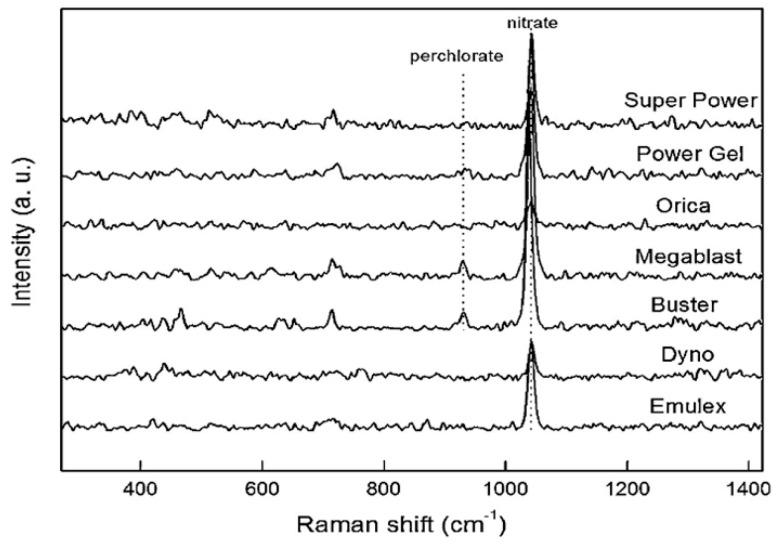
Raman spectra of the bulk volume of seven explosives. Adapted from [116], with permission from © 2013 Elsevier.

Nuntawong et al. carried on the Raman detection directly on some points on the bulk explosive samples, the analysis results indicated that the Raman signals were relatively weak and uneven [116]. Figure 12 showed the obtained Raman spectra of the bulk explosives with corresponding fluorescence baselines. Despite the fluorescent peak intensities were quite high, medium peak intensities of nitrate due to a symmetric N-O stretching were appeared at 1054 cm^−1^ for all the spectra.

A new method using confocal Raman microscopy was adopted for the measurement and recognition of microscopic post-blast particles of explosives. The results showed that the explosive mixture based on the oxide salt can successfully identify unreacted particles by confocal Raman microscopy after the explosion. Nevertheless, it was impossible to detect non reactive particles after explosion for pure organic explosives through this method [117].

Zhang et al. obtained the Raman signal intensities of ammonium nitrate, potassium nitrate, and sodium nitrate in solid samples in a distance range from 2 m to 10 m [118]. The Raman spectra of these three samples look similar: each of them has a highest peak in the vicinity of 1050 cm -1, small differences can serve as a "signature" for discriminating between them.

Recently, Hufziger et al. conducted an experiment by using a novel ultraviolet (UV) Raman standoff wide-field imaging spectrometer for detecting explosive residues [119]. The estimate detection limits of PETN and NH_4_NO_3_ is 1 mg/cm^2^ while the standoff distance is 2.3 m.

Surface-enhanced Raman scattering (SERS) technology can enlarge intensities of the Raman signals by several orders while molecules were scattered at or near hot spots, making ultrasensitive detection of samples possible, even at the single-molecule level [120]. In a recent review, the recognition of explosives in different states was summarized, the potential of SERS for vapor detection of explosives through SERS sensors as chemical noses was shown, and the problems of perchlorate anion detection in water was reviewed [121]. Hakonen et al. discussed the possible use of SERS for convenient in-situ threat recognition and summed up existing SERS detection methods and substrates with a distinctive focus on ultra-sensitive real-time detection [122]. SERS technology could achieve the detection limit of 5 ppb on a polished gold film substrate with the analysis time of 30 s for DNT. The main concepts, detection abilities, and prospects were discussed to guide homeland security and counter-terrorism. 

Jamil et al. demonstrated a rapid and selective recognition of TNT by SERS using 6-aminohexanethiol (AHT) as a novel identification molecule [123]. AHT:TNT complex formation was verified by the development of a pink color and the emergence of new band near 500 nm in UV-vis spectrum. Solution Raman spectroscopy result also supported the AHT:TNT complex formation by obvious changes in the vibrational stretching of the AHT molecule between 2800 and 3000 cm^−1^. Their method also displayed excellent selectivity towards TNT over 2,4-DNT and picric acid.

In a recent review, several detection strategies based on SERS for detecting different explosive compounds in different environments were summarized. The results indicated that explosives can be recognized in the ppb range in air as well as in the picomolar range in water. The majority of the SERS measurement procedures possess a preferable detection range compatible with applications [124].

Ultraviolet resonance Raman spectroscopy (UVRRS) has been used to examine nitro aromatic compounds (1,2-DNB, 1,3-DNB, 1,4-DNB, and TNT). The spectra showed remarkable resonance enhancement with laser wavelengths of 229 nm and 262 nm. At these wavelengths, there was a good correlation between the magnitude of enhancement and the intensity of UV-Vis absorption. This change provided another way to identify the spectra of these related substances [125].

**Figure 13:**
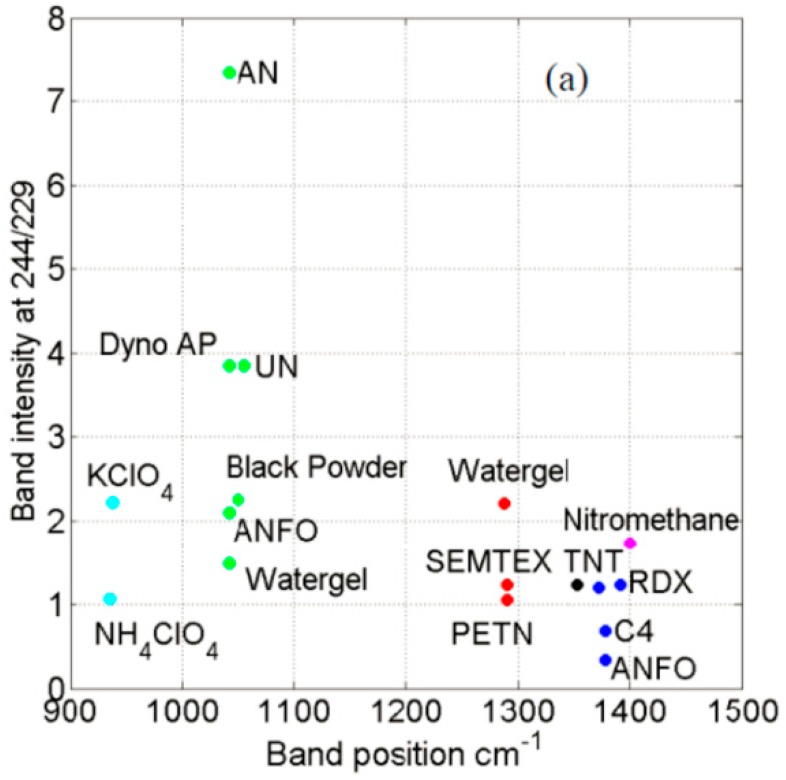
Dual-excitation-wavelength signatures of measured explosives. Adapted from [126], with permission from © 2013 SPIE.

Yellampalle et al. proposed the concept of multiple-excitation-wavelength deep-ultraviolet resonance Raman technique (DUVRRS) for the trace detection of explosives [126]. Three explosives, ammonium nitrate (AN), ammonium nitrate/fuel oil (ANFO), and Watergel containing nitrates had a very distinctive characteristic nitrate band at 1042 cm^−1^ by using the 248 nm excitation wavelength, as shown in Figure 13. ANFO had an obvious frequency band at 1378 cm^−1^, probably caused by naphthalenic-type derivatives in fuel oil. Spectral features contained sufficient variation among the intensities of the bands in each spectrum. This ensured that the three spectra could be clearly distinguished.

## 5. Ion Mobility Spectroscopy (IMS)

**Figure 14:**
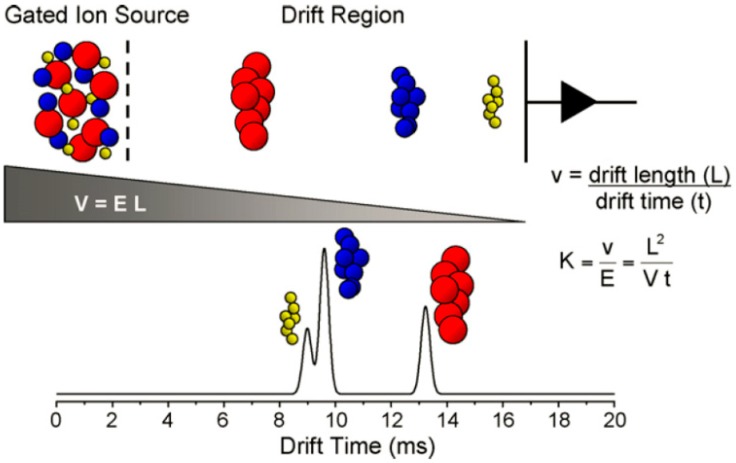
Principle of ion mobility spectroscopy. Adapted from [127], with permission from © 2007 Elsevier.

IMS is a widely used method for rapid detection of explosives at airports around the world. It has been considered to be one of the best technologies to detect trace explosives because of its low detection limit, fast response, simplicity, and strong portability [6]. The disadvantages of IMS were limited linear dynamic range and relative poor resolution, which were due to a finite reservoir of charge and limited drift tubes. The principle of typical IMS instruments is depicted in Figure 14 [127]. The sample molecules are ionized with a corona discharge source or a radiation source. The ions then come into a field-free drift region driven by an electric gradient, where they finally drift toward a collector. The compounds are identified based on the time needed for ionized molecules to drift through the electric field.

Sivakumar et al. developed an ion mobility spectrometer to detect explosive species and reached a configuration providing good sensitivity with adequate resolution [128]. TNT and RDX were detected to less than 1 ppb by volume levels in the vapor mode. The sensitivity was about 10 ng for TNT and RDX and 50 ng for PETN.

Lee et al. analyzed five commonly used explosives (RDX, HMX, TNT, DNT, and PETN) by using ion mobility spectrometry-mass spectrometry (IMS-MS) [129] RDX·NO_3_^−^, TNT^−^, PETN·NO_3_^−^, HMX·NO_3_^−^, and DNT^−^ were detected and the average drift times were 6.93 ms, 10.20 ms, 9.15 ms, 12.24 ms, 11.30 ms, and 8.89 ms, respectively. The detection limits were 0.1 ng for RDX, 10 ng for TNT, 0.5 ng for PETN, 5.0 ng for HMX, and 10 ng for DNT. They proposed that different ionization sources might cause different results.

Langmeier et al. performed the detection of TNT and HMX with a new laser ion mobility spectrometer (LIMS); detection limits were 1 ng for TNT and about 20 ng for HMX [130]. The analysis of desorption curves allowed additional spectral features to be extracted, which could be used to distinguish substances with a similar drift time.

**Figure 15:**
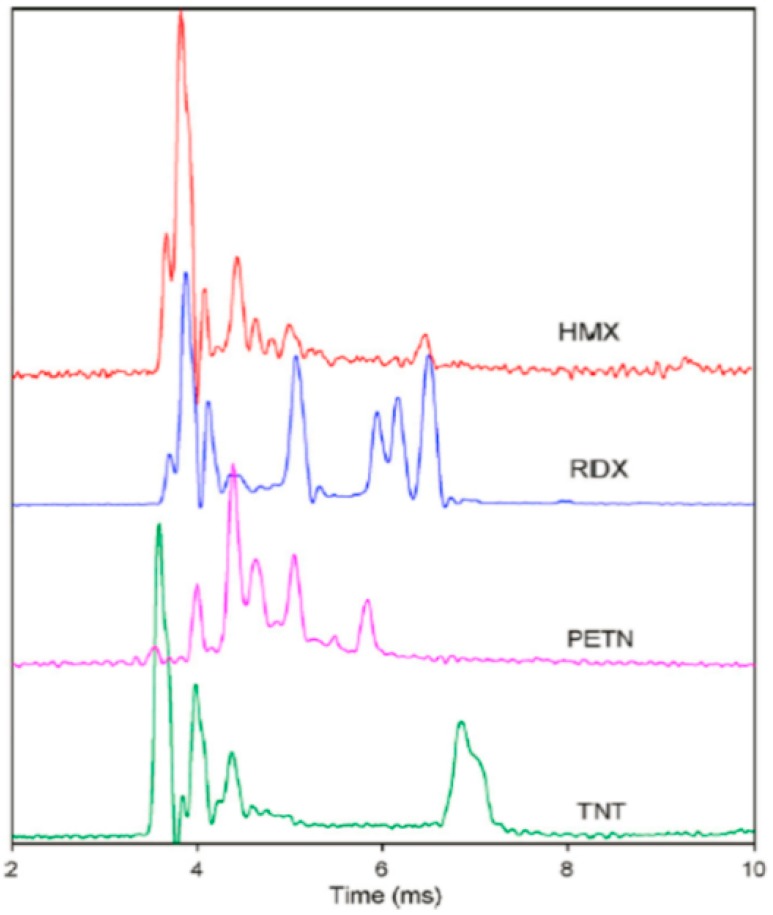
Positive ion mobility spectra of TNT, RDX, HMX, and PETN. Adapted from [131], with permission from © 2010 Elsevier.

Tabrizchi et al. used IMS to detect explosives in the positive mode, and their results were demonstrated in Figure 15 [131]. Each explosive compound has a unique pattern and displays additional peaks that can be used to identify. The detecting limit for RDX, HMX, PETN, NTO, and TNT were about 1, 10, 40, 1000, and 1000 ng, respectively.

**Figure 16:**
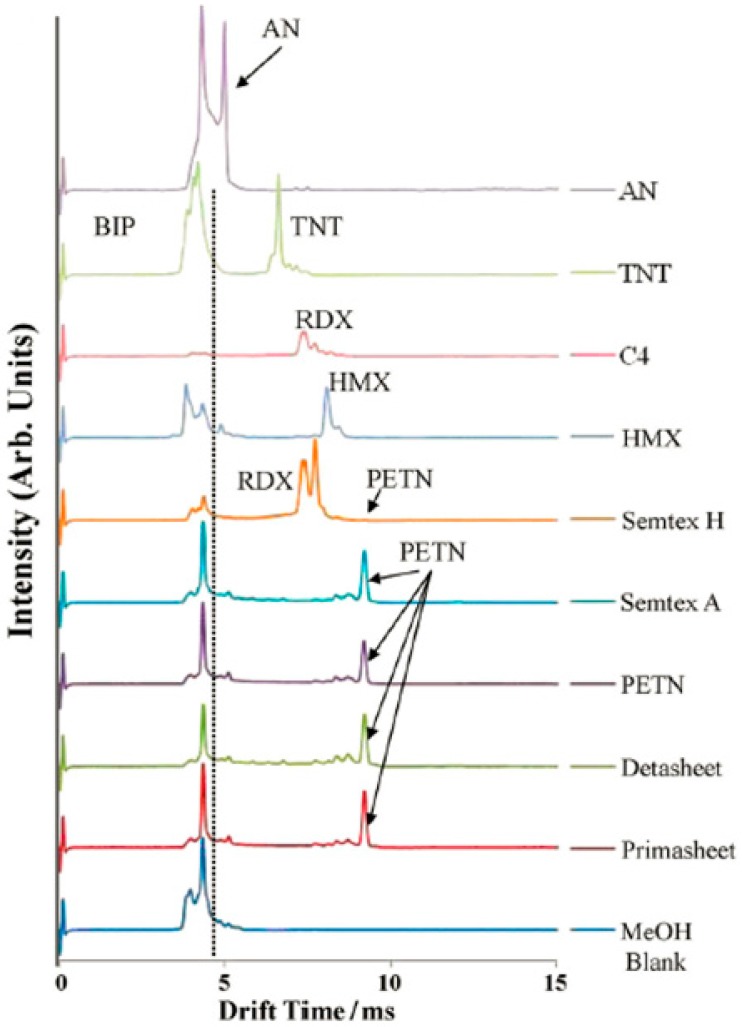
Ion mobility spectra of different explosives. Adapted from [132], with permission from © 2010 Elsevier.

Hilton et al. analyzed ten kinds of commonly used explosives with a commercial electrospray ionization-high resolution ion mobility spectrometer (ESI-HRIMS) [132]. The obtained ion mobility spectra possessed a great deal of recognition information, as shown in Figure 16. They suggested that the high resolution instrument could produce quantitative or semi quantitative information on the explosive compounds of conventional explosives sample analysis.

Solid phase micro-extraction (SPME) was coupled with IMS as a sample pre-concentration system to improve the detection of explosive residues in open areas [133]. The detection ability for DNT and TNT was obtained. Nitrocellulose (NC) spectra could also be measured by the SPME-IMS system on a reliable basis. The results indicated that this technique was useful as a potential screening tool for energetic compounds.

## 6. Conclusions and Future Prospects

The detection of explosives and related compounds has become a high priority issue in recent years for security reasons. From this review, it can be conducted that considerable progress has been achieved in different spectroscopic techniques (THz spectroscopy, LIBS, Raman spectroscopy, and IMS) for detecting various explosives. Comparisons of several properties are given in Table 4, based on referenced papers as well as subjective assessments. Additionally, there are thousands of explosive compounds and no single approach is capable of detecting every such compound. 

The use of terahertz technology to detect explosives from various sources has attracted much interest. However, so far these attempts have only been partially successful. One major reason is that current terahertz spectroscopy techniques allow the collection of spectra in a narrow terahertz window, only up to 3–6 THz. In this range, many compounds may appear similar; this window is not sufficient to distinguish the different explosives and their matrix separately. A wider terahertz bandwidth is expected to identify the salient features in the acquired spectrum unique to each molecule. Since the reflection of hazardous materials is weak and the dependence on the surface quality is strong, this measurement requires a more sensitive arrangement and careful signal processing to achieve reliable results. To avoid the problem of peak broadening, a waveguide-based TDS technique can be used in which the crystal plane is highly oriented to the emitted THz radiation.

In order to make explosive detection more reliable, LIBS still has some difficulties to overcome. As mentioned earlier, when an attempt is made to detect explosives, interference from atmospheric oxygen and nitrogen is a problem, and a system designed to prioritize remnants can help eliminate peripheral interference. Moreover, the dual pulse LIBS can reduce the contribution of the ambient atmosphere.

Further research through the use of portable Raman microscopes will also be useful so that testing whether the explosive particles can be carried out in the battlefield from the explosive mixture is a crucial aspect of the military actions. Actually, the current portable Raman spectrometer usually does not combine the microscope, so they are only applicable to the detection of massive unexploded explosives.

Ion mobility spectrometry is a widely used method for detecting explosives before detonation. It has the advantages of firmness, portability, and on-site use. Nevertheless, the research work carried out in the analytical laboratory has certain limits: low resolving power, limited selectivity, and chemical interference, so they are not always directly applicable to the comprehensive screening of various explosives. For the ion mobility spectrometry, future development directions are miniaturization, substitution of non-radioactive ionization sources, and improvement of instrument performance.

The combination of various methods is an effective way to ensure accurate detection. By sharing lasers, spectrometers, and optical paths, a combination of different spectral methods (such as Raman and LIBS) can be implemented in a single instrument, and various efforts are required to reduce the size and complexity of the instrument [134,135]. In addition, new analytical methods need to be developed to detect explosives faster and more sensitively. The technique of explosive detection is ideal for rapid real-time analysis of the minimum quantity of explosives with high precision and resolution without involving the preparation of complex samples. The continuous development of these testing technologies will result in new or even more precise applications, and the demand for the fast and reliable detection of energetic materials is increasing. At present, the Spectroscopy-based detection techniques are the most advanced in the field of explosion analysis. Therefore, it is foreseeable that they will also play a crucial role in the future. Improving the sensitivity and specificity of explosives detection technology are important principles. 

## Figures and Tables

**Table 1:** Absorption peaks of nitrogen compounds, oxidizers, and mixtures.

Compound	Measured Resonance/THz	Theoretical Resonance/THz
5-ATN	0.73, 1.23, 1.79	1.12, 1.66
BMDATHBT	1.50, 1.68	1.48, 1.70
SrNO_3_	1.80	1.72
5-ATN/SrNO_3_	0.72, 1.23, 1.80	-
BMDATHBT/SrNO_3_	1.55, 1.67, 1.80	-
KClO_3_	2.38	2.40
KClO_3_/Al/S	2.35	-
KClO_4_	2.00, 2.17	2.00, 2.20
KClO_4_/Al/S	1.96, 2.20	-

**Table 2:** List of various atomic and molecular peaks in the LIBS spectra of NTO, RDX, and HMX obtained using fs and ns pulses.

Peak/nm	Species	RDX		HMX		NTO	
		ns	fs	ns	fs	ns	fs
247.82	C	*	*	*	*	*	*
279.49, 280.21, 285.10	Mg	*	*	*	*	*	*
385.01	CN	#	*	#	*	#	*
385.40	CN	*	*	*	*	*	*
386.16	CN	*	*	*	*	*	*
387.07	CN	*	*	*	*	*	*
388.28	CN	*	*	*	*	*	*
393.25, 396.77	Ca	*	*	*	*	*	*
394.4, 396.15	Al	*	*	*	*	*	*
588.89, 589.50	Na	*	*	*	*	*	*
656.2	H	*	*	*	*	*	*
742.2, 744.1, 746.8	N	**	*	**	*	**	*
777.20	O (triplet)	*	*	*	*	*	*
818.34, 818.64, 821.50, 822.35,	N	*	*	*	*	*	*
824.22, 859.5, 856.74, 862.9, 865.66, 868.03, 870.25, 871.10, 870.74	N	**	*	**	*	**	*
844.55	O	*	*	*	*	*	*
867.80, 868.80	N	*	*	*	*	*	*

* Indicates peak present, ** indicates higher magnitude, # indicates buried in the noise.

**Table 3:** Description of elemental and molecular peaks obtained in three atmospheres for NTO, RDX, and TNT samples.

Species	Peaks/nm	NTO			RDX			TNT		
		Air	N_2_	Ar	Air	N_2_	Ar	Air	N_2_	Ar
C	247.82	*	*	*B	*	*	*B	*	*	*B
Ca	393.35, 396.83, 422.67	*	*	*	*	*	*	–	–	–
CN-(*∆**ν*** = +1)	359.02	*	*	*	*	*	*L	*	*	*L
CN-(*∆**ν*** = 0)	388.28, 387.07, 386.16, 385.40, 385.01	*	*B	*	*	*B	*	*	*B	*
CN-(*∆**ν*** = −1)	421.50, 419.63, 418.03, 416.78	*	*	*	*	*	*L	*	*	*
C_2_-(*∆**ν*** = +1)	473.63, 471.50	–	–	–	–	–	–	*	*L	*
C_2_-(*∆**ν*** = 0)	516.47	–	–	*L	–	–	*L	*	*	*B
Na	588.89, 589.50	*	*	*	*	*	*	–	–	–
Hα	656.2	*	*	*B	*	*	*	*	*	*B
O	777.2, 844.55	*B	*	*	*B	*	*	*B	*	*
N	821.50, 822.35	*	*	*	*	*	*	*	*	*
N	867.80, 868.80	*	*	*	*	*	*	*	*	*

* Indicates the presence of peak, – indicates the absence of peak, L refers to peak presence with lower intensity (or buried in the noise), B refers to peak presence with higher intensity among three atmospheres.

**Table 4:** Comparison of the four spectroscopic detection technologies.

Methods	Sample Type	Sensitivity	Molecular Selectivity	Acquisition Time	In Situ Monitoring
THz spectroscopy	condensed	medium	good	fast	Yes
LIBS	condensed	low	medium	fast	Yes
Raman spectroscopy	condensed	medium	good	slow	Yes
IMS	gas	high	medium	medium	Yes
